# A Material-by-Design Approach to Develop Ceramic- and Metallic-Particle-Reinforced Ca-α-SiAlON Composites for Improved Thermal and Structural Properties

**DOI:** 10.3390/nano12132176

**Published:** 2022-06-24

**Authors:** Hasan Sohail Syed, Abba Abdulhamid Abubakar, Abbas Saeed Hakeem

**Affiliations:** 1 Mechanical Engineering Department, King Fahd University of Petroleum and Minerals (KFUPM), Dhahran 31261, Saudi Arabia; s201729970@kfupm.edu.sa; 2 Interdisciplinary Research Center for Advanced Materials (IRC-AM), King Fahd University of Petroleum and Minerals (KFUPM), Dhahran 31261, Saudi Arabia; 3 Interdisciplinary Research Center for Hydrogen and Energy Storage (IRC-HES), King Fahd University of Petroleum and Minerals (KFUPM), Dhahran 31261, Saudi Arabia; ashakeem@kfupm.edu.sa

**Keywords:** SiAlON, spark plasma sintering, mean-field homogenization, tool insert materials, machining

## Abstract

α-SiAlON is commonly used to machine superalloys owing to its desirable thermal and structural properties. α-SiAlON is among the crystalline forms of SiAlON and has more favorable properties than β-SiAlON. However, it becomes fragile during the machining of hard-to-cut materials due to its low fracture toughness and machinability. Recent research efforts focus on improving the thermal and structural properties of α-SiAlON using suitable dopants, nano-sized precursors, and the addition of metallic/ceramic reinforcement particles. The present study presents a material-by-design approach to designing and developing ceramic and metal-particle-reinforced Ca-α-SiAlON composites with properties tailored for the cutting tool applications. The mean-field homogenization theories and effective medium approximations implemented in an in-house code are used to effectively optimize the thermal and structural properties of the Ca-α-SiAlON composite by varying essential parameters such as inclusion material, volume fraction, porosity, particulate size, and thermal interface resistance. Individual properties of the matrix and reinforcements are considered in the computations of effective properties such as thermal conductivity, thermal expansion coefficient, modulus of elasticity, and fracture toughness. The main objective of the study is to enhance the thermal conductivity and fracture toughness of Ca-α-SiAlON, while lowering its thermal expansion coefficient. At the same time, the elastic modulus and hardness/strength must be maintained within an acceptable range. As a validation, Ni/Ca-α-SiAlON and SiC/Ca-α-SiAlON composites are synthesized from the nano-sized precursors, CaO dopant, and Ni/SiC microparticles via spark plasma sintering (SPS) process. The thermal conductivity, coefficient of thermal expansion, and elastic modulus of the composites are measured and compared with the computational predictions. The computational predictions are found to be comparable to that of the experimental measurements. Moreover, the studies show that WC, SiC, and Cr can be suitable reinforcement materials for enhancing the thermal and structural properties of Ca-α-SiAlON material for the cutting tool inserts.

## 1. Introduction

Owing to its excellent thermomechanical and tribological characteristics, SiAlON has become a suitable ceramic material for a wide range of industrial applications, such as bearings, heat insulators, sealing elements, engine parts, cutting tools, refractories, etc. [1,2,3]. The use of this material originated in the early 1970s when Oyama and Kamigaito [4] and Jack and Wilson [5] reported that Al^3+^ could be infused into the lattice of Si_3_N_4_ without altering the crystal structure, i.e., by replacing Si^4+^ such that the N^3−^ are also replaced by O^2−^, to form the solid solution, SiAlON. SiAlON is thus formed from the chemical reaction involving silicon nitride (Si_3_N_4_), aluminum oxide (Al_2_O_3_), and aluminum nitride (AlN), in which the Al-N and Al-O bonds replace some of the Si-N bonds [5]. SiAlON primarily exists in four representative phases or combinations, i.e., β-SiAlON, α-SiAlON, O-SiAlON, and X-SiAlON [6,7]. β-SiAlON is the low-temperature phase commonly represented as Si_6−z_Al_z_O_z_N_8−z_, where z is the number of Si and N atoms that are replaced by the Al and O atoms; the z value ranges between 0 to 4.2 [8,9]. β-SiAlON has elongated grains; thus, it exhibits high fracture toughness. α-SiAlON is the high-temperature phase that is developed by doping with a rare-earth or an alkaline metal oxide which can be designated as M_xc_Si_12−(m+n)_Al_m+n_O_n_N_16−n_, where x = m/c, c is the valence of the metal, M stands for the rare-earth or alkaline metal [10]. It is more favorable for engineering applications because of its equiaxed grains and higher hardness, erosion strength, oxidation resistance, wear, and lubricating properties than its β counterpart [11]; however, its fracture toughness and machinability are relatively poor. 

To improve its fracture toughness and thermal shock resistance, the metal composition of the metallic oxide compound (M) is altered such that the dopant might result in both α and β-phase within the SiAlON microstructure, which becomes favorable for some practical applications [12,13]. The O-SiAlON belongs to the orthorhombic group with the stoichiometric composition Si_2−x_Al_x_N_2−x_O_1+x_ with 0.04 ≤ x ≤ 0.4. It has the lowest density and highest oxidation resistance among the various forms of SiAlON. The X-SiAlON possesses a structure like that of mullite in which tetrahedral units of AlO4 and SiO4 bridge the octahedral Al_2_O_6_ columns. It has a stoichiometric composition that varies from SiAlO_2_ to Si_16.9_Al_22.7_O_48.8_N_11.6_. However, apart from the phases mentioned earlier, there exist other polytypes of AIN, named according to Ramsdell notation as 8H, 15R, 12H, 21R, 27R and 2H polytypes; thus, SiAlON is a versatile ceramic material with complex chemistry and a high potential for various industrial applications [14,15].

Since it was found in the 1970s, the main drawbacks of SiAlON are its poor machinability and high production cost [1,2,3]. SiAlON can become too hard and brittle to the extent that machining becomes more feasible via the electrical discharge machining (EDM) process [16]. Even with the EDM, significant enhancement of the SiAlON electrical properties is critically required. Furthermore, the production of SiAlON through conventional sintering was challenging until the 2000s, when Chen and Wang [17] developed the two-step sintering method for ceramic materials. The method was used to develop several oxide and carbide ceramic composites (including SiAlON matrix composite) with controlled microstructure and enhanced mechanical properties [18,19,20,21]. More recently, spark plasma and microwave sintering became the best powder metallurgy route for synthesizing and developing SiAlON composites with better mechanical properties and microstructure morphology [22,23,24,25]. Spark plasma sintering (SPS) uses pulsed electrical current and applied pressure to sinter the powder particles at relatively lower temperatures and shorter processing time. In contrast, microwave sintering (MS) uses microwave radiation to sinter the ceramic powder with relatively more rapid and volumetric heating than SPSMS resulting in lower sintering time [26]. SiAlON has relatively lower thermal conductivity and fracture toughness as compared to other ceramic materials used for cutting tools applications. This hinders its heat dissipation ability and service life during metal cutting operations. Furthermore, manufacturing SiAlON-based tools has become very costly due to their extremely high hardness. Therefore, improving its thermal conductivity, electrical conductivity, fracture toughness and thermal expansion coefficient has become necessary for its successful use as a cutting tool material.

Several recent studies [27,28,29] have shown that SiAlON’s thermal and mechanical properties become enhanced via the reinforcement with metallic and ceramic particles to form a SiAlON matrix ceramic composite rather than the synthesizing the combined α/β-phase form. For instance, adding a 20–40 wt% ZrN inclusion to the β-Sialon matrix significantly improves fracture toughness, wear properties, and electrical conductivity, but its hardness and strength become degraded [18,30]. The addition of up to 20 wt% of TiN inclusions resulted in increased hardness, fracture toughness, electrical conductivity, and wear resistance in SiAlON. However, the strength of the nanocomposite was considerably reduced due to the high interfacial mismatch strain developed [31,32]. Similarly, the hardness and fracture toughness of SiAlON can be improved by adding cBN inclusions [33] as well as WC particles [34]. As the thermal conductivity of SiC is significantly higher than that of SiAlON, adding 10–25 wt% SiC improves the thermal diffusivity of α-SiAlON by ~30–70%. At the same time, the thermal diffusivity of β-SiAlON improves by about ~19% [35]. The thermal diffusivity increases with increasing SiC particles due to increasing the path for phonon transport. A recent study showed that the mechanical properties of SiAlON become further improved with increasing SiC particle size [36,37,38]. On the other hand, the addition of metallic inclusions (such as Ni) can improve the thermal conductivity and fracture toughness, even though the hardness and the thermal expansion coefficient become affected due to higher porosity and partial melting of metallic particles during sintering [39]. 

Although the SiAlON-based composite has been extensively studied in previous research, there are no comprehensive studies investigating the influence of a wide range of particle reinforcement types, volume fractions, and particle sizes to date. This impedes the progress of utilizing SiAlON-based composites as a replacement for pure SiAlON or other ceramic materials for various industrial applications. Therefore, there is a need for a critical evaluation of the influence of metallic and ceramic particles on the effective properties of SiAlON-based composites. For a more systematic study, computational design tools have become necessary to minimize the costs associated with the trial-and-error approach utilized experimentally.

Previous studies show that the CaO additive resulted in a single-phase α-SiAlON ceramic with a relatively more stable/dense microstructure and a suitable range of thermo-mechanical properties compared to when the other additives were used [15]. In addition, CaO is highly soluble, cheaper, and widely available from mineral resources such as fly ash; hence, Ca-α-SiAlON is chosen for the current study. The present study used a computational design approach to tailor Ca-α-SiAlON composite properties for cutting tool applications by reinforcing it with a wide range of metal and ceramic particles. Effective medium and mean-field homogenization schemes implemented in our in-house codes were used to optimize the effective properties of Ca-α-SiAlON composites to obtain superior properties that suit the intended application. The predicted properties included thermal conductivity, coefficient of thermal expansion, modulus of elasticity, and fracture toughness. The effect of the inclusion volume fraction, particle sizes, thermal interfacial resistance between the inclusion and matrix, and porosity are studied. The parameters are optimized for the thermal conductivity, fracture toughness, and thermal expansion coefficient of the Ca-α-SiAlON composite while maintaining the modulus of elasticity at an acceptable level. 

The most suitable inclusion materials were selected based on the optimized predictions from the computational studies. To validate the predictions, the designed particle volume fractions and particle sizes were used to synthesize Ni/Ca-α-SiAlON and SiC/Ca-α-SiAlON composites from the nano-sized precursors, CaO dopant, and Ni/SiC microparticles via the spark plasma sintering (SPS) process. The microstructural morphologies of the synthesized samples were assessed via scanning electron and optical microscopy, while phase characterization was conducted via X-ray diffraction analysis. Porosity was measured via the Archimedes technique, while the hardness and elastic modulus were measured using the instrumented indentation. The thermal conductivity and coefficient of thermal expansion were measured using the thermal and thermo-mechanical analyzers, respectively. The measured properties of the composite materials were then compared with that of the computational predictions.

## 2. Computational Material Design

The computational design of composite materials has become feasible with the recent advancement in computing and homogenization theories [40]. Composite materials contain certain microstructural constituents (such as inclusions, fibers, defects, pores, and interfaces) that must be selected and dispersed to attain the desired properties. Computer simulations can be used to assess the thermomechanical response of composite materials under various combinations and forms of microstructural constituents before their synthesis and development; hence, they serve as cost-effective design tools for tailoring the desired properties.

### Mean-Field Homogenization Using Effective Medium Theories

Homogenization theories are used to estimate the effective properties of heterogeneous materials through computational modeling or effective medium approximations. Effective medium theories (EMTs) are used to predict effective properties by utilizing various analytical or theoretical relations that describe the macroscopic behavior of composite materials. With EMTs, acceptable relations (or approximations) are used to predict the microscopic behavior of a composite system based on the individual properties and volume fractions of individual microconstituents. Subsequently, the macroscale response of the composite material is obtained by averaging the microscale results obtained with the theoretical relationships. 

**Figure 1:**
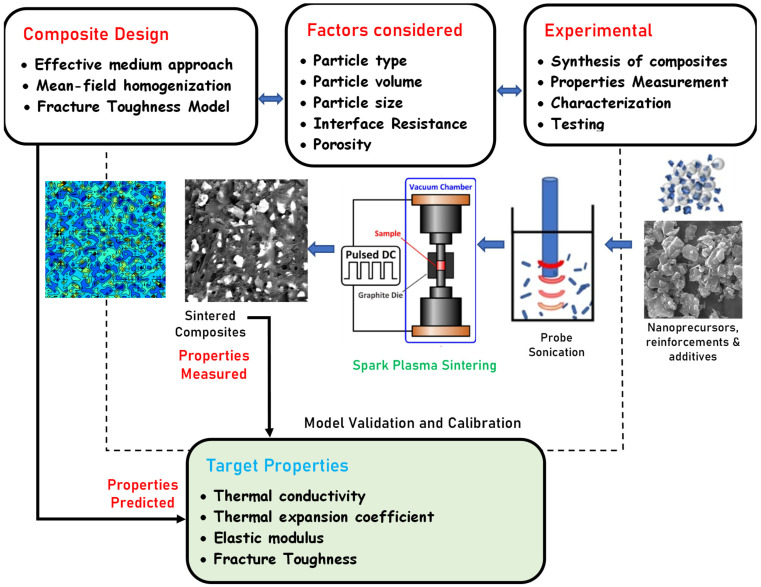
Steps highlighting the computational approach and experimental route taken in the design and development of SiAlON-based composites.

In the present study, multi-inclusion effective medium theories were used to predict the thermo-mechanical properties of various composite materials (Figure 1). The properties of the matrix and inclusion materials, particle size and shape, particle orientation, volume fraction, and interfacial thermal resistance were adopted in the calculations. The effective thermal conductivity of the composite material was predicted using the multi-inclusion effective medium approximation (EMA) model by Siddiqui and Arif [41] as well as theoretical models by Hasselman and Johnson [42]. The elastic modulus and coefficient of thermal expansion were predicted using the Mori–Tanaka mean-field homogenization scheme. In contrast, fracture toughness was predicted using the energy-based semi-empirical model previously developed by Li and Zhou [43,44].

The Hasselman and Johnson model [42] is among the earliest analytical models used to compute the effective thermal conductivity ($\kappa_{e}$) of a composite material by taking into account the thermal boundary resistance between the matrix and reinforcement phases as well as the other essential composite parameters such as particle shape, particle radius, and volume fractions (Equation (1)). The model has been proven to give accurate predictions for a composite with dilute fractions of the reinforcement material [45]. However, to apply the model to a hybrid composite with the second inclusion and/or any porosity, it is necessary to use the two-step calculation procedure where the porosity or second inclusion is used to arrive at the effective conductivity for the matrix phase [46]:

(1) $$\kappa_{e}=\frac{\kappa_{mat}^{eff}\left[2\kappa_{m}+\kappa_{inc}^{eff}+2\left(\kappa_{inc}^{eff}-\kappa_{mat}\right)\varphi_{i}\right]}{2\kappa_{mat}^{eff}+\kappa_{inc}^{eff}-\left(\kappa_{inc}^{eff}-\kappa_{mat}^{eff}\right)\varphi_{i}}$$

where $\kappa_{mat}^{eff}=2k_{m}\frac{1-\varphi_{p}}{2+\varphi_{p}}$ is the effective thermal conductivity of matrix material, $k_{m}$ is the matrix thermal conductivity, $\varphi_{i}$ is the volume fraction of the first inclusion, $\varphi_{p}$ is the porosity/second inclusion fraction, $\kappa_{inc}^{eff}=\frac{k_{inc}^{i}}{1-\frac{R_{TB}^{i}\;k_{inc}^{i}}{a^{i}}}$ is the effective thermal conductivity of the first inclusion, $k_{inc}^{i}$ is the thermal conductivity of the inclusion, $R_{TB}^{i}$ is the thermal boundary resistance, and $a^{i}$ is the inclusion radius.

Similarly, the multi-inclusion effective medium approximation (EMA) model [41] assumes that: (i) the inclusion volume fraction is small and sufficiently dilute, (ii) heat transfer across the imperfect mating interfaces is affected by the interfacial thermal resistance, (iii) porosity is the second phase inclusion with a specific size, distribution, shape, and volume fraction. The advantages of the EMA model over the Hasselman and Johnson model are the considerations for (i) nanosize effect, (ii) multiple inclusions, (iii) different particle orientation and shape, and iii) non-uniform dispersion of inclusions. Nevertheless, both theories are developed based on the concept of Kapitza radius, which represents the minimum threshold particle radius beyond which the thermal boundary resistance becomes negligible in the calculations of the effective thermal conductivity. The multi-inclusion EMA model can be expressed as [40]:

(2) $$\kappa_{e,11}=\kappa_{e,22}=\kappa_{mat}\frac{2+\sum_{i=2}^{N}\varphi^{i}\left[\beta_{11}^{i}\left(1-l_{11}^{i}\right)\left(1+〈\cos^{2}\theta {〉}^{i}\right)+\beta_{33}^{i}\left(1-l_{33}^{i}\right)\left(1-〈\cos^{2}\theta {〉}^{i}\right)\right]}{2-\sum_{i=2}^{N}\varphi^{i}\left[\beta_{11}^{i}l_{11}^{i}\left(1+〈\cos^{2}\theta {〉}^{i}\right)+\beta_{33}^{i}l_{33}^{i}\left(1-〈\cos^{2}\theta {〉}^{i}\right)\right]}$$

(3) $$\kappa_{e,33}=\kappa_{mat}\frac{1+\sum_{i=2}^{N}\varphi_{i}\left[\beta_{11}^{i}\left(1-l_{11}^{i}\right)\left(1-〈\cos^{2}\theta {〉}^{i}\right)+\beta_{33}^{i}\left(1-l_{33}^{i}\right)〈\cos^{2}\theta {〉}^{i}\right]}{1-\sum_{i=2}^{N}\varphi_{i}\left[\beta_{11}^{i}l_{11}^{i}\left(1-〈\cos^{2}\theta {〉}^{i}\right)+\beta_{33}^{i}l_{33}^{i}〈\cos^{2}\theta {〉}^{i}\right]}$$

(4) $$l_{11}^{i}=l_{22}^{i}=\{\begin{array}{c} \frac{{\left(p^{i}\right)}^{2}}{2\left({\left(p^{i}\right)}^{2}-1\right)}-\frac{p^{i}}{2{\left({\left(p^{i}\right)}^{2}-1\right)}^{1.5}}\cosh^{-1}p^{i},\;\mathrm{for}\;p^{i}\geq 1\\ \frac{{\left(p^{i}\right)}^{2}}{2\left({\left(p^{i}\right)}^{2}-1\right)}+\frac{p^{i}}{2{\left(1-{\left(p^{i}\right)}^{2}\right)}^{1.5}}\cosh^{-1}p^{i},\;\mathrm{for}\;p^{i}<1 \end{array}$$

(5) $$\begin{array}{ccc} \kappa_{c,11}^{i}=\{ & \kappa_{inc}^{i}/\left(1+\gamma_{11}^{i}L_{33}^{i}k_{inc}^{i}/k_{mat}\right),\text{ for platelet} & \kappa_{inc}^{i}/\left(1+\gamma_{11}^{i}L_{11}^{i}\kappa_{inc}^{i}/\kappa_{mat}\right),\text{ for other shapes}\\ k_{c,33}^{i}=\{ & \kappa_{inc}^{i}/\left(1+\gamma_{33}^{i}L_{11}^{i}k_{inc}^{i}/k_{mat}\right),\text{ for cylindrical} & \kappa_{inc}^{i}/\left(1+\gamma_{33}^{i}L_{33}^{i}\kappa_{inc}^{i}/\kappa_{mat}\right),\text{ for other shapes} \end{array}$$

(6) $$\gamma_{kk}^{i}=\{\begin{array}{c} \alpha_{k}\left(2+1/p^{i}\right),\;\mathrm{for}\;p^{i}\geq 1\\ \alpha_{k}\left(1+2p^{i}\right),\;\mathrm{for}\;p^{i}<1 \end{array}$$

where: $〈\cos^{2}\theta {〉}^{i}=\frac{\int^{\;}\rho^{i}\left(\theta \right)\cos^{2}\theta \sin\theta d\theta }{\int^{\;}\rho^{i}\left(\theta \right)\sin\theta \;d\theta }$ is the orientation factor for the inclusion *i*, $p^{i}=a_{3}^{i}/a_{1}^{i}$ is the particle aspect ratio, $l_{33}^{i}=1-2l_{11}^{i}$, $\beta_{kk}^{i}=\frac{\kappa_{c,kk}^{i}-\kappa_{mat}}{\kappa_{mat}+l_{kk}^{i}\left(\kappa_{c,kk}^{i}-\kappa_{mat}\right)}$, $a_{k}^{i}$ are the radii that define the particle shapes, $a_{kap}=R_{TB}^{i}\kappa_{mat}$ is the Kapitza radius, and $\alpha_{k}^{i}=a_{kap}/a_{k}^{i}$ is a dimensionless quantity that represents the influence of thermal boundary resistance on heat flow, $R_{TB}^{i}$ is thermal interface resistance, $\kappa_{mat}$ is the matrix thermal conductivity, $\kappa_{e}$ are the overall thermal conductivities along the three orthogonal axes, $\kappa_{inc}^{i}$ is the thermal conductivity of the inclusion *i* and $\varphi_{i}$ is the volume fraction of inclusions.

Eshelby’s research in 1965 provides the fundamental framework for predicting effective stiffness for inhomogeneous elastic media. According to Eshelby, the microstrain distribution developed within inhomogeneous elastic media relates to the macroscale strains via the strain localization equation. Based on Eshelby’s theory, several schemes are used to predict the effective properties of composite materials. Among other schemes, the Mori–Tanaka scheme optimally fits heterogeneous materials with inclusions (of volume fraction <30%), which may interact with one another. Using the Mori–Tanaka mean-field homogenization scheme [47], the effective elastic modulus and thermal expansion coefficient are estimated using the following equations [48]:

(7) $$C_{e}=\varphi_{i}C_{i}:A_{i}+\left(1-\varphi_{i}\right)C_{mat}:A_{mat}$$

(8) $$\alpha_{e}=\alpha_{i}I_{2}+\varphi_{i}\left(C_{i}^{-1}-C_{mat}^{-1}\right)W{\left(\left(1-\varphi_{i}\right)I_{4}+p_{i}W\right)}^{-1}$$

(9) $$A_{mat}={\left[\left(1-\varphi_{i}\right)I_{4}+\varphi_{i}B_{a}\right]}^{-1}$$

(10) $$A_{i}=B_{a}:A_{mat}$$

(11) $$B_{a}={\left[I_{4}+S:C_{mat}^{-1}\left(C_{i}-C_{mat}\right)\right]}^{-1}$$

(12) $$W=C_{i}A_{i}C_{mat}^{-1}$$

where C refers to the elastic modulus, α is the thermal expansion coefficient, A is the strain localization tensor, φ is the volume fraction, S is the Eshelby tensor, $I_{2}$ is a second-order identity tensor and $I_{4}$ is the fourth-order identity tensor. The inclusion and matrix are designated with the subscripts i and mat, respectively.

In their previous works, Doghri and Tinel [49] showed that the direct application of the Mori–Tanaka scheme for composites with dual inclusions could lead to misleading results. Hence, a modified scheme where the composite material is subdivided into pseudo-grains that contain only one type of inclusion was used in the current work. As shown in Equation (13), the required properties are more accurately estimated by applying the Voigt method to carry out homogenization across the entire grains:

(13) $${\bar{C}}_{e}=\sum_{i=1}^{N}\frac{c_{i}^{i}}{1-c_{mat}}C_{e}^{i}$$

The dominant fracture mechanism in composite materials includes particle cracking, matrix cracking, and interface debonding. The effective fracture toughness ($K_{IC}$) of the ceramic composite is predicted by the effective elastic moduli (${\bar{C}}_{e}$) and critical energy release rates ($J_{ic}$) of the ceramic composite based on Equations (14) and (15). The critical energy release rate is predicted using the analytical model by Akhtar et al. [50], which was developed based on the original model by Li and Zhou [36,37] for the prediction of fracture toughness in ceramic composites. The developed models assume fracture by quasi-static crack growth, and the ultra-low crack growth rate is predicted through some dynamic calculations. Hence, the evaluated fracture toughness depends on essential microstructural features such as inclusion material type, inclusion size and distribution, individual fracture toughness of matrix and inclusion materials, interfacial fracture energy, etc. Details about the fracture toughness model are given in the work of Akhtar et al. [50]:

(14) $$K_{IC}^{2}=J_{IC}\frac{{\bar{C}}_{e}}{1-{\bar{v}}^{2}}$$

where $\bar{v}$ is the effective Poisson’s ratio of composite system.

Due to cracking by multiple modes, the critical energy release rate is expressed as the average energy release rate due to particle-cracking, matrix-cracking, and interface-debonding:

(15) $$J_{IC}=\xi \left(s,f\right)\left(\varphi_{int}H_{int}+\varphi_{mat}H_{mat}+\varphi_{i}H_{i}\right)$$

where $\xi \left(Q,\;s,f\right)=\frac{1-e^{D_{2}}}{e^{D_{1}}-e^{D_{2}}}e^{D_{1}f}+\frac{e^{D_{1}}-1}{e^{D_{1}}-e^{D_{2}}}e^{D_{2}f}$ is the crack length multiplication factor, $D_{1}$ and $D_{2}$ are functions of inclusion particle sizes, φ is the fracture energy, H is the proportional crack length, in denotes the inclusion material, mat denotes the matrix, and i denotes the inclusion particle. The proportional crack lengths can be estimated from the empirical relations:

(16) $$H_{int}=\frac{\int_{0}^{D}P_{ij}\left(D\right)dx}{D}p{\left(\frac{\varphi_{in}}{\varphi_{mat}}\right)}^{-a\ln Q}$$

(17) $$H_{i}=\left(\frac{\int_{0}^{D}P_{ij}\left(D\right)dx}{D}\left(1-p\right)+\frac{\int_{0}^{D}P_{jj}\left(D\right)dx}{D}\right){\left(\frac{\varphi_{in}}{\varphi_{mat}}\right)}^{-b\ln Q}$$

(18) $$H_{mat}=1-H_{int}-H_{i}$$

where D is the total crack length, p is the probability of crack deflection, Q is the strength ratio and $P_{ij}$ is probabilistic functions for matrix and particle cracking.

The Ca-α-SiAlON properties are enhanced by adding a single inclusion from various materials, sizes, and distributions to the ceramic matrix to attain better cutting performance and tool life. The desired properties considered include high thermal conductivity, low coefficient of thermal expansion (CTE), moderate elastic modulus, and high fracture toughness. Table 1 highlights the properties of Ca-α-SiAlON and the various combinations of metallic and ceramic inclusion materials considered for the present study. Three metallic inclusion materials, including Ni, Co, and Cr, were selected due to their high fracture toughness and high electrical and thermal conductivities. However, compatibility with the ceramic material becomes an issue due to the high structural mismatch at mating interfaces resulting from the considerable variation in CTE between the metallic reinforcement and ceramic matrix materials. With the use of ceramic filler materials (such as the those provided in Table 1), the structural mismatches at the mating interfaces do not usually occur; hence, ceramic inclusion materials that possess relatively higher electrical and thermal conductivity are selected for the present study (i.e., WC, SiC, ZrB_2_, cBN, and TiN). The main issue with the ceramic class is their low fracture toughness, even though their dispersion within the ceramic matrix is expected to increase the crack growth resistance.

## 3. Experimental

Following the computational design of the composite materials, a few Ca-α-SiAlON-matrix composites were reinforced with the SiC and Ni particles that were synthesized for validation. The details describing the materials, synthesis, testing, and characterization are discussed in the following sections.

### 3.1. Materials and Synthesis Details

The chemical composition of the Ca-α-SiAlON matrix was Ca_0.8_Si_9.2_Al_2.8_O_1.2_N_14.8_, corresponding to m = 1.6 and *n* = 1.2 in the general formula for Ca-α-SiAlON, i.e., Ca_m/v_Si_12−(m+n)_Al_m+n_O_n_N_16−n_. The starting precursors used in preparing the Ca-α-SiAlON matrix were composed of nano-sized precursors: Silicon dioxide (SiO_2_, 10~20 nm, Sigma Aldrich, St. Louis, MO, USA), aluminum nitride (AlN, <100 nm, Sigma Aldrich, St. Louis, MO, USA), α-crystalline silicon nitride (Si_3_N_4_, ~150 nm, UBE Industries, Minato-ku, Tokyo, Japan), amorphous silicon nitride (Si_3_N_4_, ~150 nm, UBE Industries, Minato-ku, Tokyo, Japan), calcium oxide (CaO, ~160 nm, with purity >99%, Sigma Aldrich, St. Louis, MO, USA), and Aluminum oxide (Al_2_O_3_, ~150 nm, Chempur, Karlsruhe, Germany). As for the inclusion particles, silicon carbide (SiC, ~11 µm, Buehler, Lake Bluff, IL, USA) and Ni powder (<100µm, 99.99% Fisher Scientific, Pittsburgh, PA, USA) were used. SiC was reduced in size by ball milling (using Union Process HD01/HDDM, Akron, OH, USA) in an ethanol medium for 3 h at 1000 rpm. The powder-to-balls ratio was 1:20 for ZrO_2_ balls with an average diameter of 625 µm. Based on five observations acquired from a Microtrac particle size analyzer (Model S3500/Turbotrac, York, PA, USA), the resulting particle size was estimated at about 2 µm.

To validate the computational predictions, SiC (as a ceramic reinforcement) and Ni (as a metal reinforcement) were selected to synthesize Ca-α-SiAlON matrix composites with varying contents (0 wt%, 10 wt%, 20 wt%, 30 wt%) in line with the computational predictions. To prepare the powder mixtures for each sample, the starting precursors were carefully weighed. Table 2 shows the details of the prepared composites. Each sample’s powder blends were placed in a methanol medium and then dispersed for around 20 min by ultrasonic probe sonicator (VCX 750, Sonics, Newtown, CT, USA). After homogenization, the samples were placed in a muffle furnace for around 24 h at a temperature of 80 °C for drying. After they had dried, the homogenized mixtures were crushed using a granite pestle and mortar for ease of consolidation. Subsequently, the homogenized mixtures were consolidated in a 20 mm graphite die for 30 min at 1500 °C, 100 °C/min rate, and a uniaxial pressure of 50 MPa by SPS (HP D5, FCT Systeme, Gewerbepark, Frankenblick, Germany). Experiments were conducted at a pressure of 5 × 10^−2^ mbar. To remove the graphite embedded on the surface of the synthesized samples, the samples were ground using SiC abrasive paper with grit sizes ranging from 60 to 1000 grit sizes. Moreover, an alumina suspension (particle size of 9 μm) was used to obtain mirror-like surfaces.

### 3.2. Characterization and Testing Details

The 18 mm synthesized samples had their thermal conductivities measured at room temperature by applying continuous heat using a one-sided interfacial heat reflector (TCi, C-Therm, Fredericton, NB, Canada). A diamond cutter was used to cut each of the four samples into three pieces, one of which was utilized to measure the coefficient of thermal expansion (using TMA/SDTA 1 L.F./1100, Mettler Toledo, Columbus, Ohio, Switzerland). The Vickers hardness of the SiAlON samples was determined using instrumented indentation (ZHU250, ZwickRoell, Horsham, Pennsylvania, Germany) at a load of 10 kg. The backscattered mode of a field emission scanning electron microscope (FESEM, FEI, Hillsboro, OR, USA) was used to examine the microstructure of the samples. It was equipped with an energy-dispersive X-ray spectrometer (EDX, Malvern Panalytical, Worcester, MA, USA), which aided in collecting compositional data. The Archimedes principle was used to determine the density of the sintered samples.

The Evans and Charles Equation (Equation (19)) was used to compute the fracture toughness (*K_IC_*), where *H* is the Vickers hardness, *a* is the length from the center of the indentation to its edge, and *c* is the length from the end of the vertical crack line to the end of the horizontal crack line.

(19) $$K_{Ic}=0.16{\left(\frac{c}{a}\right)}^{-1.5}\;\left(H\times a^{0.5}\right)$$

## 4. Results and Discussion

### 4.1. Computational Prediction of Effective Properties

Computational prediction of the effective properties is based on effective medium approximations for various combinations of inclusion materials, volume fractions, and porosity utilizing the various material properties obtained from the literature. This section summarizes the findings of the computational study conducted with the mean-field homogenization codes. Firstly, the effective properties were predicted for selected reinforcement materials and several combinations of inclusion volume fraction, size, and porosity to identify the threshold for real improvement in the required properties. The porosity and inclusion particle size were optimized, while the volume fraction range was determined. The inclusion particle size is solely optimized utilizing the thermal conductivity code. Simultaneously, the porosity is fixed at a particle level based on the results of effective thermal conductivity, elastic moduli, and CTE. The final stage of the computational study involved running the simulations for varying volume fractions, fixed porosity, and particle sizes to determine the effective properties resulting from the addition of each inclusion material shown in Table 1. The data generated at this stage was used to hone the effective composite properties and identify the most suitable inclusion materials to improve cutting tool life and performance.

**Figure 2:**
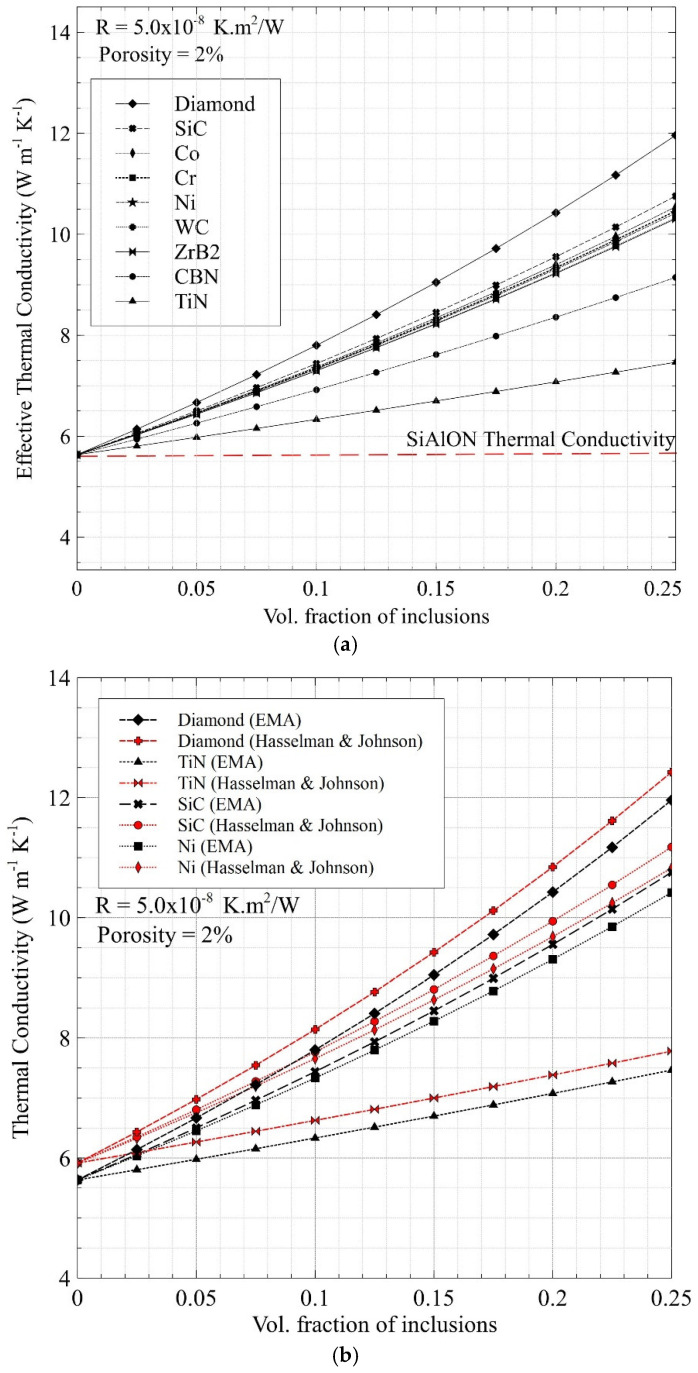
(**a**) Predicted effective thermal conductivity of Ca-α-SiAlON composites as a function of volume content of potential metal and ceramic reinforcements. (**b**) Comparison of predictions made with the modified EMT and Hasselman–Johnson models. The porosity is assumed to be 2% and the thermal interface resistance (*R*) is taken as 5.0 × 10^−8^ K.m^2^/W.

Figure 2 illustrates the effective thermal conductivities predicted for the Ca-α-SiAlON composites as a function of the volume fractions of several ceramic and metal reinforcements following Table 2. Overall, it can be observed that the thermal conductivity increases proportionally to the volume fractions, i.e., as the volume fraction of the inclusions increases, the thermal conductivity of the composite increases because of the filler material having higher thermal conductivity. The diamond-reinforced composite had the most significant rise in thermal conductivity, followed by the cluster of SiC, Co, Cr, Ni, WC, ZrB_2_, and finally, cBN and TiN. The rise in the thermal conductivity due to the addition of diamond inclusion is the highest because it has the highest thermal conductivity (~2000 W/m.K). TiN, predictably, displays the lowest rise in the composite’s thermal conductivity, which can be attributed to it having the lowest thermal conductivity (~19 W/m.K) among the remaining inclusions. Hence, by considering the highest and the lowest thermal conductivity rises as exceptional cases or outliers, the region of the aforementioned cluster is deemed the most appropriate to aid in the selection of the most optimal effective thermal conductivity (~8 W/m.K) with respect to its volume fraction (~16%). 

It is worth mentioning that the aforementioned thermal conductivity trends were obtained at a fixed interfacial resistance (ITR) value of 5.0 × 10^−8^ K.m^2^/W, a porosity of 2%, and a particle size of 10 µm. The average value for the ITR between the inclusions and Ca-α-SiAlON matrix is 5.0 × 10^−8^ K.m^2^/W as obtained from the literature [51]. The ITR value between the inclusion and SiAlON matrix depends on the wettability of the two phases, which can be controlled by proper functionalization for any desired value. Hence, it is possible to further the thermal conductivity by appropriate surface treatment of particles if deemed necessary. Although an average ITR value was selected for our model, the relationship between ITR and particle size was essential in governing the effective thermal conductivity of metal/ceramic-reinforced ceramic matrix composites. As the inclusion particle size decreased, the ITR increased because a reduction in particle size increased contacted the surface area. The Kaptza radius for the Ca-α-SiAlON composite is about 0.29 µm; thus, the thermal boundary resistance created by the 10 µm size inclusions does not notably influence the thermal conductivity values. However, the porosity and volume fractions directly affected the effective thermal conductivity. The thermal conductivity decreased with increasing porosity, which was associated with the high thermal resistance due to the reduction of gas-phase heat conduction. 

Figure 2b shows that the predictions made with the model by Hasselman and Johnson slightly overestimated the effective thermal conductivities of the composite materials. This can be attributed to the incorporation of porosity (as the second inclusion) via the two-step approach with the model by Hasselman and Johnson. On the contrary, the effective medium approximation (EMA) model implicitly considered multiple inclusions and porosity in the formulation; hence, it could lead to a better estimation of the effective thermal conductivities. Nevertheless, the predictions made with the two theories considered here were in very close range for the 10 µm particle size adopted here.

**Figure 3:**
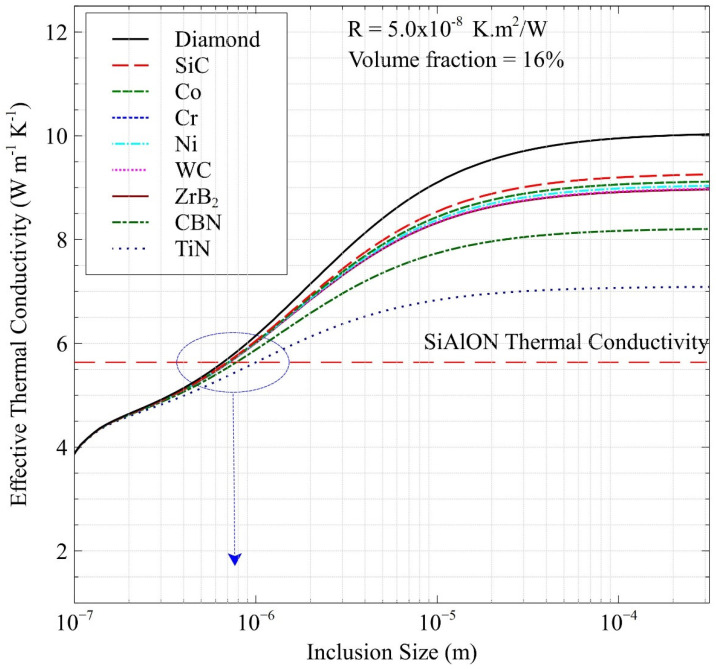
Determination of threshold particle size of reinforcements in the Ca-α-SiAlON composites for enhanced effect on thermal conductivity. Numerous metal and ceramic particles are considered with a porosity of 2%, thermal interface resistance (R) of 5.0 × 10^−8^ K.m^2^/W, and volume fraction of 16%.

Figure 3 was plotted in accordance with the determined volume fraction of 16% along with the average ITR value of 5.0 × 10^−8^ K.m^2^/W to predict the change in the effective thermal conductivity with respect to inclusion size and to determine the threshold particle size value. The general trend observed is an increase from the plain Ca-α-SiAlON thermal conductivity value of 7.6 W/mK only after an inclusion size of approximately 1 µm. This result was taken as the minimum threshold particle size post from which the ascension of thermal conductivity can be observed. Additionally, the saturation points for all the reinforced composites coincided at roughly 130 µm. Although increasing the volume fractions and reducing the porosity results in higher thermal conductivity, other effective properties, such as the elastic modulus and CTE, need to be considered for benchmarking inclusion volume fractions.

**Figure 4:**
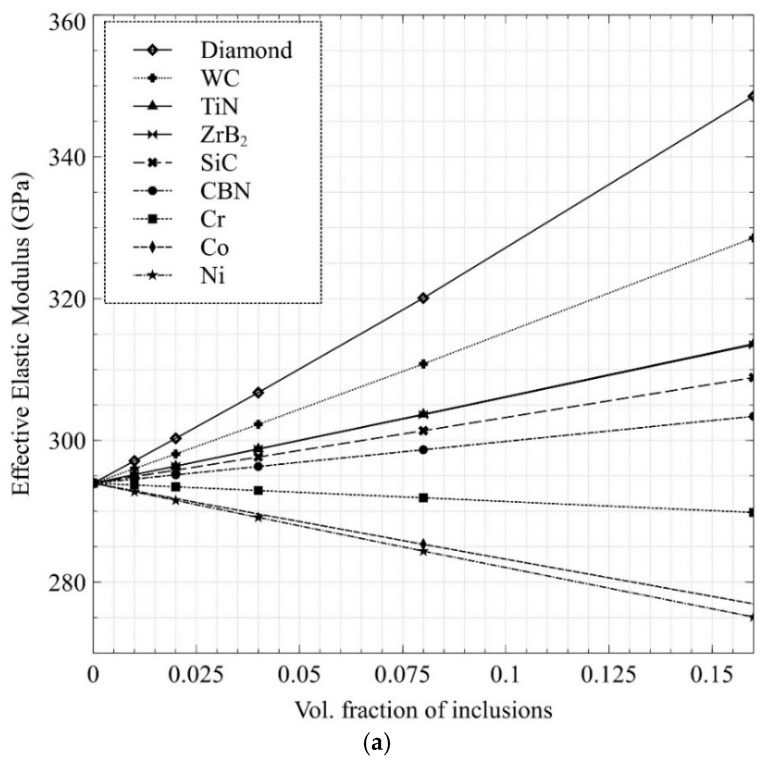

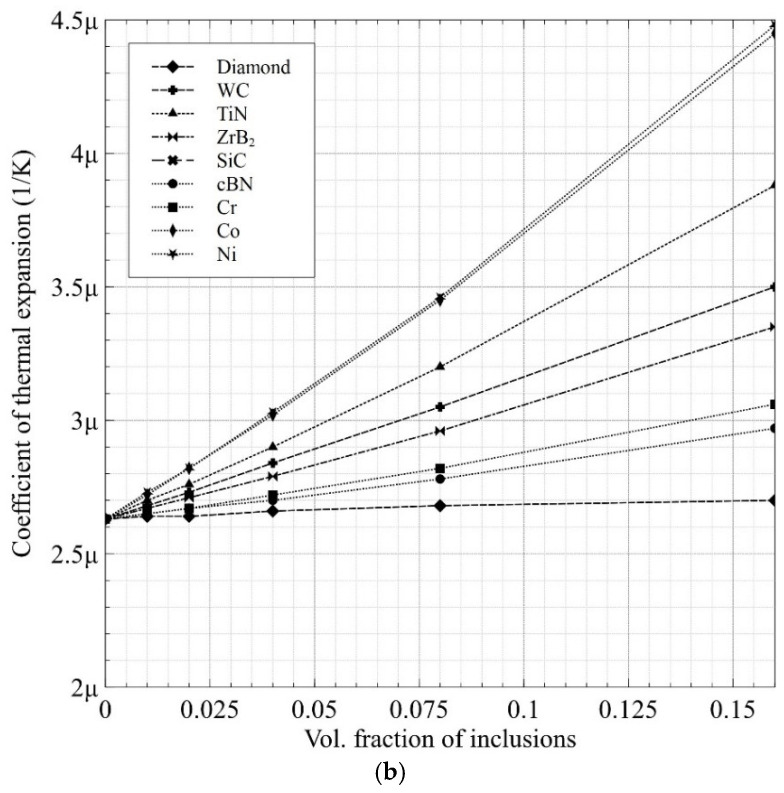
Predicted effective: (**a**) elastic modulus and (**b**) coefficient of thermal expansion of Ca-α-SiAlON composites as a function of volume content of potential metal and ceramic reinforcements.

Figure 4a depicts the predicted effective elastic modulus for various ranges of volume fractions and the reinforcement materials stated in Table 2. It can be observed that the addition of reinforcements from the cluster of diamonds through SiC increases the elastic modulus, with the diamonds providing the sharpest rise. This can be attributed to them showcasing higher elastic moduli than the plain Ca-α-SiAlON matrix. In contrast, the grouping of Cr through Ni shows a decline in the elastic modulus for the opposite reason (comparatively lower elastic modulus than plain Ca-α-SiAlON). Moreover, cBN’s elastic modulus is akin to that of plain SiAlON, so the rise in elastic modulus is only marginal.

Figure 4b shows the change in the effective coefficient of thermal expansion (CTE) with respect to the volume fraction of the nine previously mentioned reinforcements. The composite must maintain a low CTE and high thermal conductivity to ensure that the thermal loads are low and heat dissipation is high, respectively, for improved cutting tool performance. In this regard, SiC, cBN, and Cr were deemed to be the most appropriate reinforcers as they do not cause the CTE of the composite to upsurge dramatically with an increase in inclusion volume fraction. Diamond would be the best candidate in this regard, but it is ignored due to its high cost. Ni through ZrB_2_, on the other hand, can be considered unsatisfactory for our target application due to an increase in CTE with the addition of more inclusion material. It is already established that the CTE and material elastic modulus are interrelated. Materials having strong interatomic interactions, such as ceramics, have low CTEs and high elastic moduli, making them stiffer. Hence, by adding metal inclusions such as Ni and Co in Ca-α-SiAlON, which has high CTE, but low elastic modulus, the CTE is expected to increase rise, as predicted by the model, and verified experimentally.

**Figure 5:**
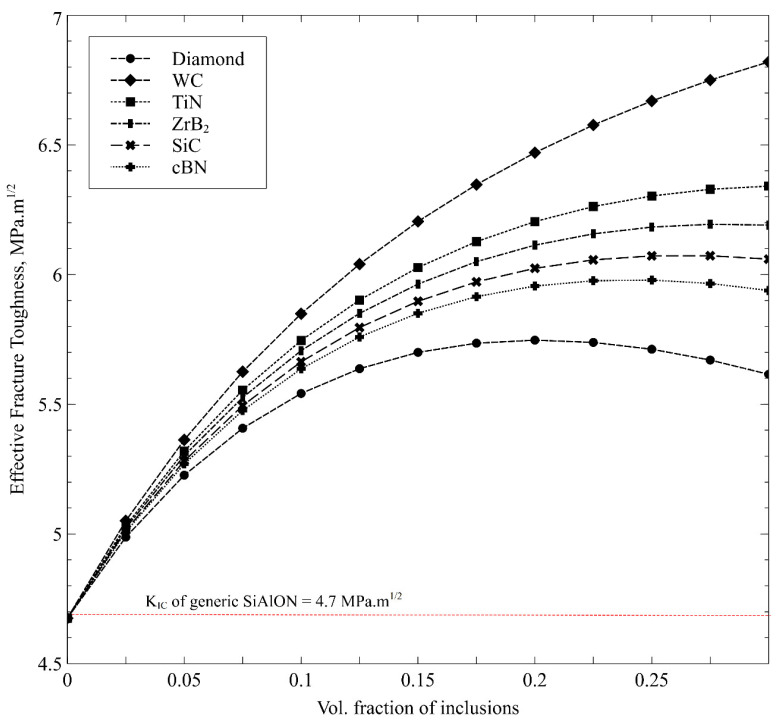
Predicted effective fracture toughness of Ca-α-SiAlON composites as a function of volume content of potential ceramic reinforcements.

At high cutting speeds, increased fracture toughness can be beneficial to the cutting tool’s durability and lifetime. The computational model is initially performed for various potential ceramic reinforcements to determine the most appropriate candidates likely to improve the effective *K_IC_* of the resulting Ca-α-SiAlON composite. Figure 5 shows the effective *K_IC_* as a function of various ceramic inclusions in the Ca-α-SiAlON matrix, assuming a particle size of 10 µm in each combination. The shape of the inclusions was assumed to be perfectly spherical, and the aspect ratio was kept constant. It is worth noting that the *K_IC_* of generic Ca-α-SiAlON [52] (~4.67 Mpa.m^1/2^, which corresponded to fracture energy of 60 J/m^2^) was set as a benchmark to assess the selected reinforcement types. Metal reinforcements are excluded from the analysis as the existing computational model for fracture toughness is valid only for ceramic particles in ceramic matrices. It can be observed that the effective *K_IC_* increases as the volume fraction of inclusions increases in general. However, *K_IC_* drops after around 20% volume for inclusions having low fracture resistance, such as diamond and cBN. This peak value of *K_IC_* shifts towards a higher volume percentage value for inclusions with higher fracture resistance. Hence, the WC with the highest fracture energy (85 J/m^2^) leads to the highest *K_IC_* with a continuously increasing trend, followed by TiN and ZrB_2_. The decreasing trend in *K_IC_* beyond a specific volume concentration (in the case of reinforcements with low fracture energies) is attributed to the dominant particulate cracking mode, unlike interface cracking at high volume percentages in the Ca-α-SiAlON matrix. As a result, this tendency shows that crack deflection, rather than particle cracking, can be fostered. Therefore *K_IC_* can be improved by including inclusions with high fracture energies for a well-bonded interface, as considered in the current model and reported by Kumai et al. [53]. As a result, particles with high fracture energy can be employed to increase the *K_IC_* of SiAlON composites.

### 4.2. Characterization and Morphology of Synthesized Samples

The interaction of reinforcements with the SiAlON matrix directly impacts the final properties such as thermal conductivity, expansion coefficient, stiffness, and fracture toughness. The accuracy of the computational predictions was validated through an experimental phase. This included determining the composite samples’ characteristics and characterization. To evaluate the computational design accuracy, a variety of SiC and Ni-reinforced Ca-α-SiAlON composites were developed in line with predictions using spark-plasma sintering. A comprehensive microscopic study of the sintered sample was performed to describe distinct composite combinations.

**Figure 6:**
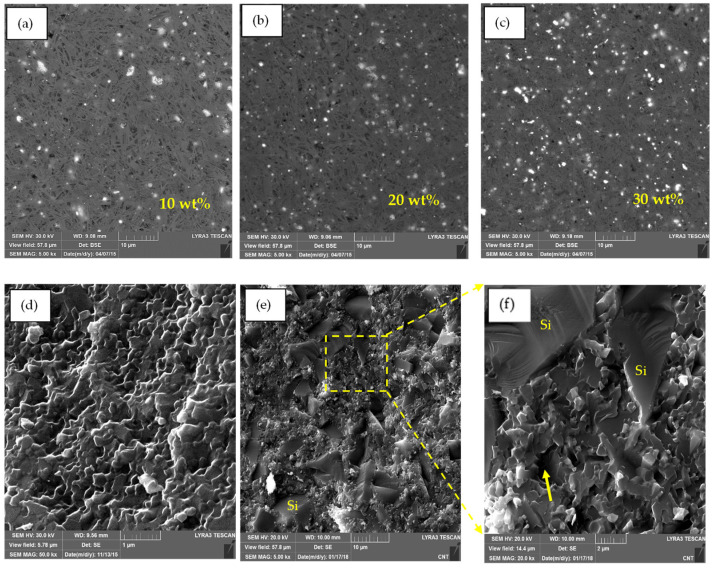
Polished FESEM images (**a**–**c**) of the Ca-α-SiAlON composites with 10 wt.%, 20 wt.%, and 30 wt.% SiC compositions, respectively (the black area shows the SiAlON matrix, and the bright dispersed phase is SiC), (**d**) FESEM image of the fracture surface morphology of the monolithic α-SiAlON, (**e**) FESEM image of 30 wt.% SiC/Ca-α-SiAlON composite (**f**) A close-up view of the image in (**e**).

FESEM images of the polished surfaces of the sintered 10% SiC, 20% SiC, and 30% SiC Ca-α-SiAlON composites are shown in Figure 6a–c, respectively. The homogenous dispersion of the white SiC particles in the black Ca-α-SiAlON matrix demonstrates that the ultrasonic probe sonication was sufficient to achieve appropriate SiC particle homogeneity. FESEM micrographs of fractured surfaces of monolithic Ca-α-SiAlON and 30%SiC/Ca-α-SiAlON composite samples are shown in Figure 6d,e, respectively. As can be observed, a typical microstructure of single-phase α-SiAlON with equiaxed grains are evident in the case of a monolithic SiAlON sample. An intergranular form of fracture in monolithic SiAlON is evident without any major pull-out of particles. On the contrary, sharp step-like structures inside the SiC/SiAlON composite indicate cleavage or delamination in the grains. Grain pull-out and cleavage stages have been identified as essential variables in improving the fracture toughness of ceramic composites with increasing SiC content [54]. 

**Figure 7:**
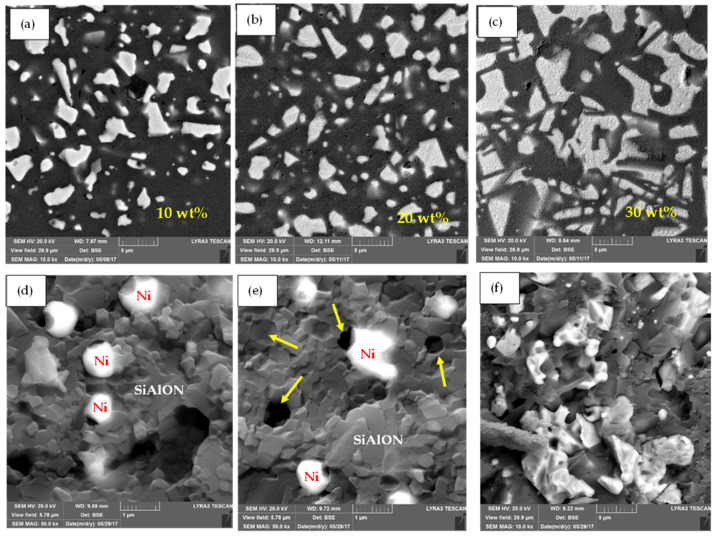
Polished FESEM images (**a**–**c**) of the Ca-α-SiAlON composites with 10 wt.%, 20 wt.%, and 30 wt.% Ni compositions, respectively (the black area shows the SiAlON matrix, and the bright dispersed phase is Ni). FESEM images (**d**–**f**) of the fracture surface morphology of the Ca-α-SiAlON composites with 10 wt.%, 20 wt.%, and 30 wt.% Ni compositions, respectively. The arrows in the figures show the voids that appeared due to the pullout of Ni particles during sample preparation.

Figure 7a–f shows FESEM micrographs of the polished and fracture surface morphologies of Ca-α-SiAlON composites-reinforced with 10 wt.%, 20 wt.%, and 30 wt.% Ni compositions. These micrographs are composed of uniformly dispersed bright Ni particles embedded in a grey Ca-α-SiAlON matrix. Some of the Ni inclusions, however, tore away from the matrix and left a void imprint, as represented by arrows in fractured morphology (Figure 7d,e). Ni particles can be seen scattered throughout the Ca-α-SiAlON matrix with uniform dispersion. The morphology of the α-SiAlON grains in Ni/SiAlON samples is equiaxed, and the addition of Ni particles did not affect the morphology of the α-grains. Moreover, a micrograph of a monolithic Ca-α-SiAlON sample (Figure 6d) showed little or no porosity compared to other SiC and Ni-containing composites (Figure 7f). 

**Figure 8:**
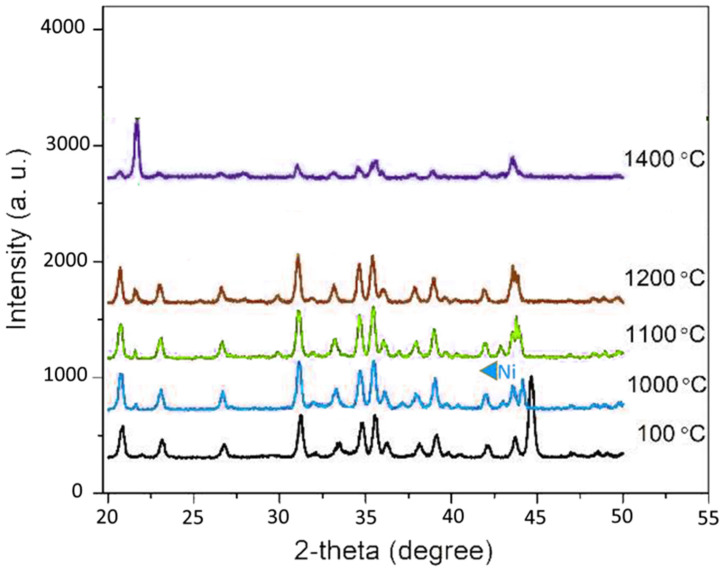
XRD pattern of 30 wt%Ni/Ca-α-SiAlON at different sintering temperatures.

Figure 8 shows the XRD pattern of the 30 wt% Ni/Ca-α-SiAlON composite at the various sintering temperatures. The mixture of the precursors was heated up to 1500 °C during the high-temperature XRD under an argon atmosphere. The crystalline phases were continually noted during the heating process. Si_3_N_4_ is the dominant constituent of the powder mixture, and a major peak of Ni was visible at 100 °C and a diffraction angle of 44.5°. The diffractogram shows that the presence of Ni reinforcements particles did not react to form any new compounds such as the aluminides, silicides, etc. However, a notable change in the XRD pattern is observed at about 1200 °C and 1400 °C with the formation of the liquid phase Ca-α-SiAlON and solution-diffusion-precipitation phenomenon, respectively. The AlN and the intermediate phase(s) disappear at temperatures above 1400 °C, while the formation of the α-sialon increases during the heating process until reaching completion at about 1500 °C.

### 4.3. Validation of Predictions

Experimental measurements of the composite properties such as thermal conductivity, CTE, hardness, and fracture toughness, were compared to the numerical predictions. The base properties of sintered Ca-doped α-SiAlONs were first determined experimentally and then used as input in the computational modeling for recalibration and realistic predictions.

The measured and predicted thermal conductivity, CTE, and hardness of monolithic Ca-α-SiAlON and 10% Ni-, 20% Ni-, and 30% Ni-reinforced Ca-α-SiAlON produced are shown in Table 3. The porosity of each sintered sample is also presented. The difference between the predicted and experimental values of thermal conductivity and CTE is less than 8%, which is deemed ab acceptable agreement. The discrepancy between experimental and numerical results can be attributed to several assumptions. The spherical shape of inclusions, uniform particle size distribution, and perfect dispersion of Ni inclusions inside the SiAlON matrix are among these assumptions. More importantly, the data on interface thermal resistance was collected from the published literature. With an increase in Ni content, the porosity of the composites was found to increase, which was also confirmed by the decrease in the relative density of the resulting composites. This decrease in density and increase in porosity is associated with the softening and/or melting of Ni particles during the composite’s synthesis process. As a result, the mismatch of the CTE between Ca-α-SiAlON matrix and Ni results in the formation of voids at the interface. Furthermore, deposits of Ni were found on the exterior of the synthesized samples, demonstrating that some of the Ni particles melted and exited the graphite die during the synthesis. As the nickel content increased, the amount of nickel that melted increased, resulting in lower relative densities. This was confirmed when the actual amount of Ni in the final composite was compared to the initial value used in calculating theoretical density.

Table 3 depicts the thermal conductivity variation as a function of Ni volume fractions within the Ca-α-SiAlON matrix which are all measured at room temperature. The thermal conductivity of the composites with 10% Ni, 20%, and 30% Ni content is somewhat higher than that of monolithic SiAlON (5.65 W/m K). However, as demonstrated, increasing the Ni level had no discernible influence on the thermal conductivity values. This is obviously due to the draining out of the Ni content and not achieving the desired Ni content, as more liquid Ni ran out of the graphite die at 1500 °C. Furthermore, the heat transfer across different phases, i.e., Ca-α-SiAlON matrix and Ni, and their interface all affect a composite’s thermal conductivity, directly related to the particle size, voids, porosity, and interface resistance between the two phases [55,56]. A similar effect of porosity is observed on the effective CTE of Ni/Ca-α-SiAlON composites when the Ni content increases from 10% to 30%. The measured CTE of 10% Ni/Ca-α-SiAlON composite (3.18 × 10^−6^/K) was found to be larger than that of monolithic Ca-α-SiAlON (2.63 × 10^−6^/K), which is due to a higher intrinsic CTE of pure Ni (13.1 × 10^−6^/K). Nevertheless, the effective CTE decreased as the Ni content increased. Because porosity tends to accommodate the material’s internal thermal expansion, CTE decreases with associated porosity. Considering measured thermal conductivity (5.65 W/m K) and CTE (2.63 × 10^−6^/K) of α-SiAlON together with observed porosity and Ni particle size of around 2µm, computational predictions are run and compared with the experimental values. A very close agreement can be found, as depicted in Table 3.

**Figure 9:**
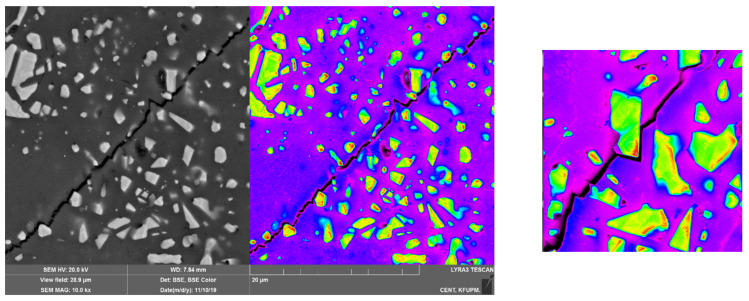
FESEM image showing crack propagation within the 20% Ni/Ca-α-SiAlON composite.

The Vickers indentation test was done on a monolithic Ca-α-SiAlON and sintered SiAlON composites with 10%, 20%, and 30 wt% Ni, with hardness values recorded decreasing from 21.1 GPa (for monolithic α-SiAlON) to the lowest value of 16.5 GPa in the case of 30%Ni/SiAlON composite, where the porosity is the highest. As expected, an increased porosity as the Ni loading increases tends to reduce the composites’ hardness. Although the elastic modulus was not experimentally measured, a complementary trend is predicted numerically. The hardness of composite tends to increase with an increase in the elastic modulus, as predicted (Table 3). Due to the mismatch in the ability to deform a ductile Ni particle and a brittle SiAlON matrix, cracks are deflected when they reach a SiAlON/Ni interface, as can be observed in Figure 9. The presence of Ni in pores/voids in the matrix were expected to promote grain bonding/wettability of Ni with Ca-α-SiAlON matrix at the interface, resulting in increased fracture toughness values due to the decrease in hardness as observed in this study.

**Figure 10:**
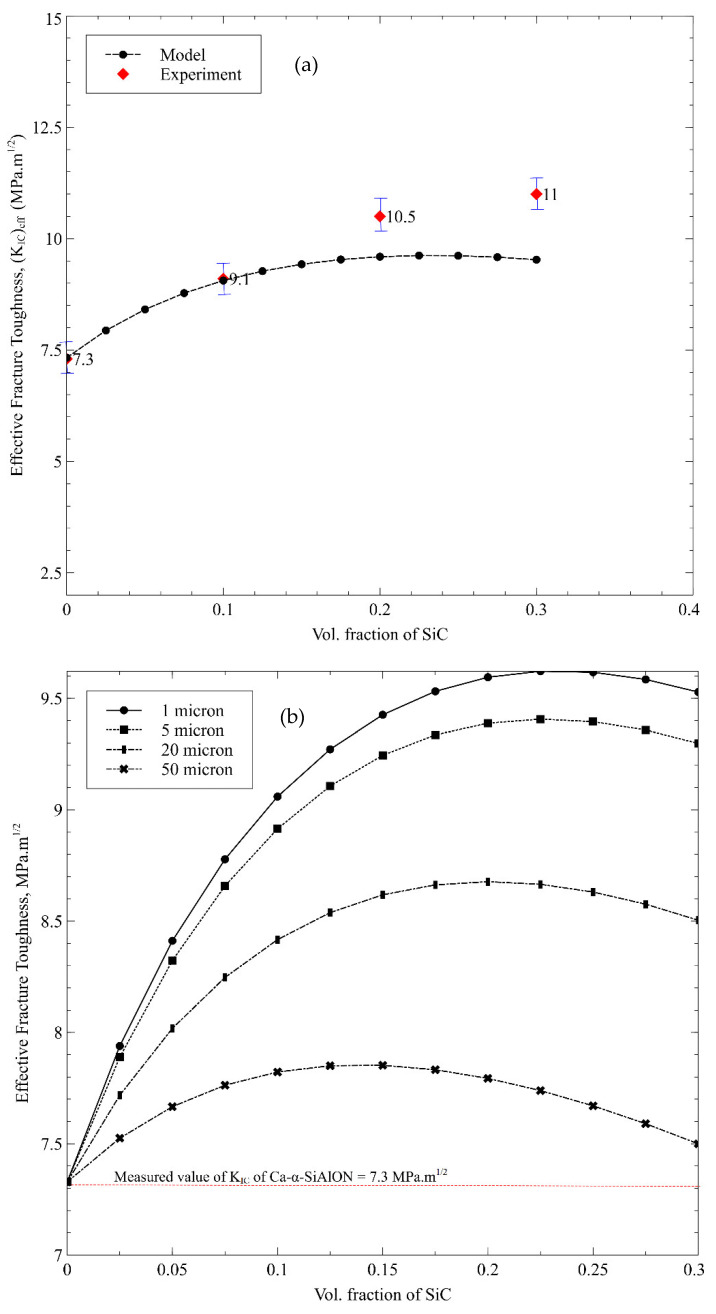
(**a**) Predicted and measured fracture toughness of the Ca-α-SiAlON/SiC composite for different compositions. A SiC particle size of 2 µm was used in synthesized composites, (**b**) Variation of effective fracture toughness of Ca-α-SiAlON/SiC composite as a function SiC particle size and volume fraction.

Figure 10a compares the measured *K_IC_* values of sintered Ca-α-SiAlON composites (with 10%, 20%, and 30% volume of SiC) with that of the model predictions. It is worth noting that a SiC particle size of 3 µm was used in this model, which is in line with the sintered SiC/Ca-α-SiAlON samples. A very close agreement can be found mainly at low SiC content. To determine the impact of inclusion volume fraction and size on fracture toughness of Ca-α-SiAlON composites, SiC is used as a reinforcement in the model with various particle sizes and volume fractions, as depicted in Figure 10b. As discussed earlier in Figure 5, a similar trend of growing and subsequently lowering KIC can be observed for a specific particle size and varying volume fractions, which shows that fracture by particle-cracking can dominate the interface-debonding with increasing volume fractions.

**Figure 11:**
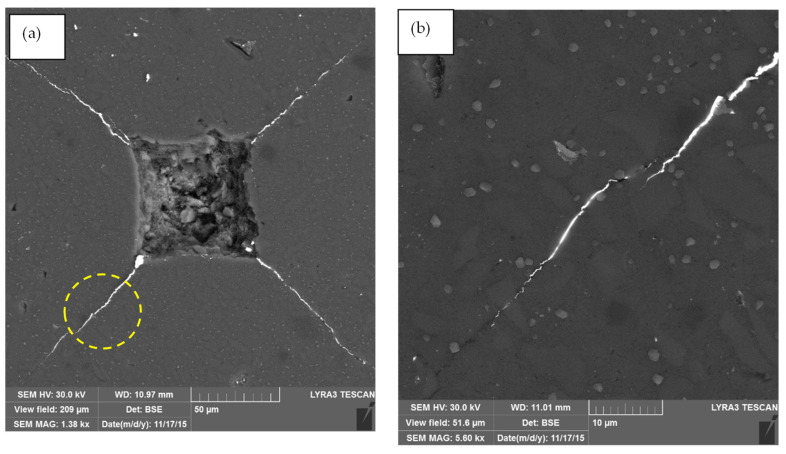
(**a**) FESEM image depicting the crack length and its propagation within 10% SiC/Ca-α-SiAlON composite. (**b**) A close-up FESEM view showing the crack propagation.

In terms of the particle size effect on *K_IC_*, it is worth noting that decreasing particle size from 50 µm to 20 µm, 5 µm, then to 5 µm enhances *K_IC_* for any given SiC content. Furthermore, as the particle size decreases from 50 µm to 1 µm, the maxima of the curves corresponding to the highest *K_IC_* increasingly shift to the right. Peak shifting implies the embedment of smaller particles in the Ca-α-SiAlON matrix due to higher particle cracking resistance for any volume content, resulting in a more considerable *K_IC_* value due to increased crack deflection. In other words, the lower *K_IC_* values of composites embedded in the Ca-α-SiAlON matrix at larger particle sizes are due to an increased tendency for particle-cracking, as also reported by Evans [57]. Due to the large surface area of smaller particles contained in the Ca-α-SiAlON matrix, crack deflections through the interface between the matrix and particle tend to promote, resulting in increased *K_IC_* FESEM image shown in Figure 11 depicts the crack length and its propagation within 10% SiC/Ca-α-SiAlON composite complementing the aforementioned reasons for enhanced fracture toughness.

## 5. Conclusions

The present study uses a material-by-design approach to develop ceramic and metal particles-reinforced Ca-α-SiAlON composites with thermal and structural properties tailored for the cutting tools application. The mean-field homogenization and effective medium theories are used to predict the effective thermal conductivity computationally, coefficient of thermal expansion, elastic modulus, and fracture toughness of Ca-α-SiAlON composites by considering various reinforcement materials, volume fractions, and particle size. The simulations were run to identify the threshold inclusion size and volume fractions that could be used to achieve the desired level of improvements in the composite properties, which might lead to enhancement in cutting performance. The computational predictions are compared with the experimental measurements conducted on the Ni/Ca-α-SiAlON and SiC/Ca-α-SiAlON composite samples. The comparison between the model predictions and the experimental data shows good agreement with respect to the thermal and structural properties of the composite.

The overall thermal conductivity of Ca-α-SiAlON ceramic improved with the addition of the selected reinforcement materials (Diamond, SiC, Co, Cr, Ni, cBN, and TiN), depending on the inclusion volume fractions and particle sizes. A particle size of 10 μm could be sufficient to enhance the thermal conductivity of Ca-α-SiAlON to the threshold level. The most significant improvement was attained for the 15% volume fractions, with the most significant improvement noted for the diamond particles (i.e., about 43%). At the same time, SiC, Co, Cr, and Ni resulted in higher values of the effective thermal conductivities within a similar range (i.e., about 33–40%). The coefficient of thermal expansion (CTE) of Ca-α-SiAlON with the volume fractions increases for the Ni, Co, and TiN reinforcements. However, the build-up of high thermal stresses during cutting operations might restrict the use of the materials mentioned earlier as suitable reinforcers for the Ca-α-SiAlON composite. In this regard, SiC, cBN, and Cr become the most appropriate reinforcers for the ceramic material. The effective fracture toughness of the Ca-α-SiAlON composite also increases with increasing reinforcement volume fractions. However, for the low fracture-resistant materials, such as diamond and cBN, the improvement in fracture toughness became poor, beyond about 20% of the volume fractions. Consequently, WC, TiN, SiC, and ZrB2 particles yielded the most remarkable improvement in fracture toughness with a continuously increasing trend of fracture resistance. SiC and WC can result in up to about 30% improvement in the overall fracture toughness. Therefore, the suitable reinforcement materials for α-SiAlON-based cutting tool inserts can be WC, SiC, and Cr due to the resulting improvement in the thermal and structural properties of the ceramic composites.

## Figures and Tables

**Table 1:** Properties of various metal and ceramic particles used in the computation design of Ca-α-SiAlON composites.

Material	Elastic Modulus (GPa)	Shear Modulus (GPa)	Bulk Modulus(GPa)	Poisson’s Ratio	Fracture Energy (J/m^2^)	CTE (10^−6^/K)	Thermal Cond. (W/mK)
Ca-α-SiAlON	306	116	200	0.27	60	2.60	5.85
Nickel (Ni)	207	79	183	0.31	-	13.1	60.1
Cobalt (Co)	211	79	193	0.32	-	12.5	69.2
Chromium (Cr)	248	110	153	0.21	-	6.20	69.1
Cubic Boron Nitride (cBN)	367	180	228	0.15	15	4.80	42.1
Silicon Carbide (SiC)	410	180	250	0.14	27	4.00	120
Tungsten Carbide (WC)	630	243	375	0.24	85	5.40	85
Titanium Nitride (TiN)	450	240	325	0.12	31.5	9.35	19.2
Zirconium Diboride (ZrB_2_)	475	215	300	0.13	24	6.20	83.8
Diamond	1000	455	539	0.29	3.52	1.18	2000

**Table 2:** Compositions (in wt.%) of SiC- and Ni-reinforced α-SiAlON composites with powder precursors.

Reinforcement Type	Reinforcement Composition (wt. %)	CaO (wt. %)	SiO_2_ (wt. %)	AlN (wt. %)	Si_3_N_4_ (wt. %)
Ni	0	7.57	2.03	19.37	71.03
	10	6.82	1.83	17.43	63.93
	20	7.06	1.62	15.500	56.82
	30	5.30	1.42	13.56	49.72
SiC	0	7.57	2.03	19.37	71.07
	10	6.81	1.83	17.43	63.96
	20	6.06	1.62	15.50	56.86
	30	5.30	1.42	13.56	49.75

**Table 3:** A comparative analysis of computational prediction and experimentally measured thermomechanical properties of Ni-reinforced Ca/α-SiAlON composites. ** The hardness of composite tends to increase with an increase in the elastic modulus as predicted. * Experimentally measured porosity from actual samples.

Property	Monolithic Ca-α-SiAlON	Ni/Ca-α-SiAlON Composites					
	Measured	10 wt%	20 wt%	30 wt%	10 wt%	20 wt%	30 wt%
		Measured			Predicted		
Porosity * (%)	2	3	10	17	Predictions are based on experimentally measured porosity *		
Thermal Conductivity (W/m-K)	5.65 (1)	5.81 (2)	5.79 (3)	5.81 (4)	6.08	5.96	5.84
CTE (10^−6^/K)	2.63 (1)	3.18 (2)	2.90 (2)	2.77 (3)	3.25	3.12	3.07
Elastic Modulus ** (MPa)	-	-	-	-	277	231	192
Hardness ** HV_10_ (GPa)	21.1 (6)	18.3 (8)	17.1 (6)	16.5 (7)	-	-	-

## Data Availability

The data presented in this study are available on request from the corresponding author.

## References

[1] Klemm H. (2010). Silicon nitride for high-temperature applications. J. Am. Ceram. Soc..

[2] Kurama S., Schulz I., Herrmann M. (2009). Wear behaviour of α-and α/β-SiAlON ceramics stabilized with Nd_2_O_3_ and Y_2_O_3_. J. Eur. Ceram. Soc..

[3] Bitterlich B., Bitsch S., Friederich K. (2008). SiAlON based ceramic cutting tools. J. Eur. Ceram. Soc..

[4] Oyama Y., Kamigaito O. (1971). Solid solubility of some oxides in Si3N4. Jpn. J. Appl. Phys..

[5] Jack K.H., Wilson W.I. (1972). Ceramics based on the Si-Al-ON and related systems. Nat. Phys. Sci..

[6] El-Amir A.A.M., El-Maddah A.A., Ewais E.M.M., El-Sheikh S.M., Bayoumi I.M.I., Ahmed Y.M.Z. (2021). Sialon from synthesis to applications: An overview. J. Asian Ceram. Soc..

[7] Narciso F.J., Rodriguez-Reinoso F. (1994). Synthesis of β-SiAlON from clays: Effect of starting materials. J. Mater. Chem..

[8] Haubner R., Herrmann M., Lux B., Petzow G., Weissenbacher R., Wilhelm M. (2003). High Performance Non-Oxide Ceramics II.

[9] Mohamedkhair A.K., Hakeem A.S., Drmosh Q.A., Mohammed A.S., Baig M.M., Ul-Hamid A., Gondal M.A., Yamani Z.H. (2020). Fabrication and Characterization of Transparent and Scratch-Proof Yttrium/Sialon Thin Films. Nanomaterials.

[10] Cao G.Z., Metselaar R. (1991). .alpha.’-Sialon ceramics: A review. Chem. Mater..

[11] Chen I.-W., Rosenflanz A. (1997). A tough SiAlON ceramic based on α-Si_3_N_4_ with a whisker-like microstructure. Nature.

[12] Richerson D.W., Lee W.E. (2018). Modern Ceramic Engineering: Properties, Processing, and Use in Design.

[13] Izhevskiy V.A., Genova L.A., Bressiani J.C., Aldinger F. (2000). Progress in SiAlON ceramics. J. Eur. Ceram. Soc..

[14] Narciso F.J., Linares-Solano A., Rodriguez-Reinoso F. (1995). Synthesis of β-sialon from coals or natural graphite. J. Mater. Res..

[15] Biswas M., Sahoo A., Muraleedharan K., Bandyopadhyay S. (2021). Crystal Structure of 27R-SiAlON Synthesized Under Carbothermal Nitridation. Trans. Indian Ceram. Soc..

[16] Kaya P., Gregori G., Yordanov P., Ayas E., Habermeier H.-U., Maier J., Turan S. (2017). An alternative composite approach to tailor the thermoelectric performance in SiAlON and SiC. J. Eur. Ceram. Soc..

[17] Chen I.-W., Wang X.-H. (2000). Sintering dense nanocrystalline ceramics without final-stage grain growth. Nature.

[18] Yin L., Jones M.I. (2020). Fabrication and properties of Sialon-ZrN composites by two-step sintering. Int. J. Refract. Met. Hard Mater..

[19] Xu T., Wang C.-A. (2016). Effect of two-step sintering on micro-honeycomb BaTiO_3_ ceramics prepared by freeze-casting process. J. Eur. Ceram. Soc..

[20] Zhang J., Zheng Y., Chen J., Zhou W., Zhao Y., Feng P. (2018). Microstructures and mechanical properties of Mo_2_FeB_2_-based cermets prepared by two-step sintering technique. Int. J. Refract. Met. Hard Mater..

[21] Zhang Z., Liu Y., Yao G., Zu G., Wu D., Hao Y. (2012). Synthesis and characterization of dense and fine nickel ferrite ceramics through two-step sintering. Ceram. Int..

[22] Canarslan Ö.S., Koroglu L., Ayas E., Canarslan N.S., Kara A., Veronesi P. (2021). Susceptor-assisted fast microwave sintering of TiN reinforced SiAlON composites in a single mode cavity. Ceram. Int..

[23] Al Malki M.M., Khan R.M.A., Hakeem A.S., Hampshire S., Laoui T. (2017). Effect of Al metal precursor on the phase formation and mechanical properties of fine-grained SiAlON ceramics prepared by spark plasma sintering. J. Eur. Ceram. Soc..

[24] Zheng L., Liu M., Zhang H., Zheng Z., Wang Z., Cheng H., Wang P., Liu Y., Huang B. (2021). Fabrication of ZnO Ceramics with Defects by Spark Plasma Sintering Method and Investigations of Their Photoelectrochemical Properties. Nanomaterials.

[25] Song Y., Liu W., Sun Y., Guan S., Chen Y. (2021). Microstructural Evolution and Mechanical Properties of Graphene Oxide-Reinforced Ti6Al4V Matrix Composite Fabricated Using Spark Plasma Sintering. Nanomaterials.

[26] Benavente R., Salvador M.D., García-Moreno O., Peñaranda-Foix F.L., Catalá-Civera J.M., Borrell A. (2015). Microwave, Spark Plasma and Conventional Sintering to Obtain Controlled Thermal Expansion β-Eucryptite Materials. Int. J. Appl. Ceram. Technol..

[27] Hakeem A.S., Laoui T., Ehsan M.A., Ahmed B.A. (2020). Spark Plasma Method for Making cBN/SiAlON Ceramic. U.S. Patent.

[28] Nekouee K.A., Khosroshahi R.A. (2016). Preparation and characterization of β-SiAlON/TiN nanocomposites sintered by spark plasma sintering and pressureless sintering. Mater. Des..

[29] Maglica A., Krnel K., Pribošič I., Kosmač T. (2007). Preparation and properties of β-SiAloN/ZrN nano-composites from ZrO_2_-coated Si_3_N_4_ powder. Process. Appl. Ceram..

[30] Yin L., Gao W., Jones M.I. (2019). Wear behaviour and electrical conductivity of β-Sialon-ZrN composites fabricated by reaction bonding and gas pressure sintering process. Ceram. Int..

[31] Li X., Yin S., Huang S., Luo H., Tang Q. (2020). Fabrication of Durable Superhydrophobic Mg Alloy Surface with Water-Repellent, Temperature-Resistant, and Self-Cleaning Properties. Vacuum.

[32] Grigoriev S., Pristinskiy Y., Volosova M., Fedorov S., Okunkova A., Peretyagin P., Smirnov A. (2021). Wire electrical discharge machining, mechanical and tribological performance of TiN reinforced multiscale SiAlON ceramic composites fabricated by spark plasma sintering. Appl. Sci..

[33] Ahmed B.A., Hakeem A.S., Laoui T., Khan R.M.A., Al Malki M.M., Ul-Hamid A., Khalid F.A., Bakhsh N. (2017). Effect of precursor size on the structure and mechanical properties of calcium-stabilized sialon/cubic boron nitride nanocomposites. J. Alloys Compd..

[34] Biswas M., Sarkar S., Halder R., Bysakh S., Muraleedharan K., Bandyopadhyay S. (2020). Sintering and characterization of a hard-to-hard configured composite: Spark plasma sintered WC reinforced α−SiAlON. J. Phys. Chem. Solids.

[35] Akin S.R.K., Turan S., Gencoglu P., Mandal H. (2017). Effect of SiC addition on the thermal diffusivity of SiAlON ceramics. Ceram. Int..

[36] Heydarian A., Abdolkarim Sajjadi S., Johnsson M. (2020). A proposed model for spark plasma sintering of SiC-Si nanocomposite with different SiC particle sizes. J. Compos. Mater..

[37] Khan R.M.A., Ahmed B.A., Al Malki M.M., Hakeem A.S., Laoui T. (2018). Synthesis of hard and tough calcium stabilized α-sialon/SiC ceramic composites using nano-sized precursors and spark plasma sintering. J. Alloys Compd..

[38] Liu L., Ye F., Zhang Z., Zhou Y. (2011). Elongation of α-SiC Particles in Spark Plasma Sintered α-SiAlON/α-SiC Composites. J. Am. Ceram. Soc..

[39] Adeniyi A.S., Ahmed B.A., Hakeem A.S., Patel F., Bakare A.I., Ul-Hamid A., Khan A.A., Ehsan M.A., Khan T.I. (2019). The Property Characterization of α-Sialon/Ni Composites Synthesized by Spark Plasma Sintering. Nanomaterials.

[40] Zhang R., Xu T., Yao B., Liu Z. (2022). Perspectives in the new era of materials intelligent design. Mater. Lab.

[41] Siddiqui M.U., Arif A.F.M. (2016). Generalized effective medium theory for particulate nanocomposite materials. Materials.

[42] Acikbas N.C. (2018). Tribological behavior of αı/βı-SiAlON-TiN composites. J. Eur. Ceram. Soc..

[43] Li Y., Zhou M. (2013). Prediction of fracturess toughness of ceramic composites as function of microstructure: II. analytical model. J. Mech. Phys. Solids.

[44] Li Y., Zhou M. (2013). Prediction of fracture toughness of ceramic composites as function of microstructure: I. Numerical simulations. J. Mech. Phys. Solids.

[45] Caccia M., Rodríguez A., Narciso J. (2014). Diamond Surface Modification to Enhance Interfacial Thermal Conductivity in Al/Diamond Composites. JOM.

[46] Molina J.-M., Rodríguez-Guerrero A., Louis E., Rodríguez-Reinoso F., Narciso J. (2017). Porosity Effect on Thermal Properties of Al-12 wt % Si/Graphite Composites. Materials.

[47] Xu Y., Tanaka Y., Goto M., Zhou Y., Yagi K. (2004). Thermal conductivity of SiC fine particles reinforced al alloy matrix composite with dispersed particle size. J. Appl. Phys..

[48] Akhtar S.S., Siddiqui M.U., Kabeer R., Hakeem A., Kareem L., Arif A.F. (2017). A computational and experimental study on the effective properties of Al_2_O_3_-Ni composites. Int. J. Appl. Ceram. Technol..

[49] Doghri I., Tinel L. (2005). Micromechanical modeling and computation of elasto-plastic materials reinforced with distributed-orientation fibers. Int. J. Plast..

[50] Akhtar S.S., Waqar T., Hakeem A.S., Arif A.F.M., Al-Athel K.S. (2019). Design and Development of Hybrid Al_2_O_3_ Based Composites with Toughening and Self-Lubricating Second-Phase Inclusions. Materials.

[51] Wang H., Xu Y., Shimono M., Tanaka Y., Yamazaki M. (2007). Computation of interfacial thermal resistance by phonon diffuse mismatch model. Mater. Trans..

[52] Munro R.G., Freiman S.W., Baker T.L. Fracture Toughness/Fracture Energy Data for Ceramics. https://srdata.nist.gov/CeramicDataPortal/ftmain.

[53] Kumai S., King J.E., Knott J.F. (1991). Fatigue in SiC-particulate-reinforced aluminium alloy composites. Mater. Sci. Eng. A.

[54] Zhou C.R., Yu Z.B., Krstic V.D. (2007). Pressureless sintered self-reinforced Y-α-SiAlON ceramics. J. Eur. Ceram. Soc..

[55] Akhtar S.S. (2022). A systematic design to develop high-performance sintered particulate copper-composite as heat spreader material. Eng. Sci. Technol. Int. J..

[56] Azam M.U., Ahmed B.A., Hakeem A.S., Irshad H.M., Laoui T., Ehsan M.A., Patel F., Khalid F.A. (2019). Tribological behaviour of alumina-based nanocomposites reinforced with uncoated and Ni-coated cubic boron nitride. J. Mater. Res. Technol..

[57] Evans A.G. (1990). Perspective on the development of high-toughness ceramics. J. Am. Ceram. Soc..

